# Downregulation of motility during stress requires stressosome input in *Listeria monocytogenes* strain EGD-e

**DOI:** 10.1128/aem.02539-25

**Published:** 2026-04-03

**Authors:** Teresa Tiensuu, Duarte N. Guerreiro, Dmitriy Ignatov, Ana H. Oliveira, Conor O'Byrne, Jörgen Johansson

**Affiliations:** 1 Laboratory for Molecular Infection Medicine Sweden, Umeå University 8075, Umeå, Sweden; 2 Department of Molecular Biology, Umeå University 8075, Umeå, Sweden; 3 Umeå Centre for Microbial Research, Umeå University 8075, Umeå, Sweden; 4 Max Planck Unit for the Science of Pathogens, Berlin, Germany; 5 Bacterial Stress Response Group, Microbiology, University of Galway, Ryan Institute, School of Biological and Chemical Sciences, Galway, Ireland; The University of Tennessee Knoxville, Knoxville, Tennessee, USA

**Keywords:** stress regulation, bacterial motility, SigB, RsbX, *Listeria monocytogenes*

## Abstract

Motility genes in *Listeria monocytogenes* are expressed under saprophytic conditions (30°C or less) but are repressed within the host at 37°C. Motility is costly on cellular resources due to the large molecular structures that need to be synthesized, and the proton motive force required for flagellar rotation. Here, we investigated the impact of the SigB-mediated general stress response on the regulation of motility in *L. monocytogenes* and sought to elucidate the regulatory steps involved. We show that an *rsbX* mutation that is unable to inactivate the stressosome, the sensory hub at the top of the SigB activation pathway, results in motility repression. Escape from this repressed state occurred through the acquisition of spontaneous mutations that decreased SigB activity. Flagellar expression was abolished in strains lacking RsbX, and a transcriptomic analysis revealed that the entire flagellar operon was strongly repressed, including the motility anti-repressor gene *gmaR*. These effects could be reversed by providing functional copies of *rsbX* or *gmaR in trans*. Abolishing expression of an antisense RNA lying opposite the large flagella operon or deleting the *mogR* transcriptional repressor restored the ability to produce flagellin in the Δ*rsbX* background. Stressosome mutations that negatively affect SigB activity resulted in a derepressed motility phenotype, whereas those that increase SigB activity resulted in a decreased motility phenotype similar to the Δ*rsbX* strain. Our data indicate that stress sensing via the stressosome negatively impacts motility and shows that the general stress response is prioritized when *L. monocytogenes* encounters osmotic and light stress conditions.

**IMPORTANCE:**

Motility involves the synthesis and operation of the flagella, which come at a high energy cost for the bacterium and need to be carefully controlled. The human pathogen *Listeria monocytogenes* senses and responds to various stresses, in part through the alternative sigma factor σ^B^ (SigB), which controls the general stress response regulon. Since the SigB-regulon harbor hundreds of genes, the activity of SigB needs to be carefully controlled under non-stressed conditions to save energy. On the other hand, upon stress, the bacterium needs to invest a large amount of energy to synthesize a myriad of proteins to cope with the increased stress. In this study, we examined how motility is regulated under osmotic and visible light stress. Our data imply that increased SigB activity negatively impacts motility gene expression by a signal that is conveyed through the stressosome multiprotein complex.

## INTRODUCTION

Flagellar motility is advantageous for bacteria, letting them move to environments richer in nutrients or away from adverse conditions. It can also promote bacterial adhesion, aid in the formation of biofilms, and for some bacteria, contribute to cellular invasion (1). However, the expression and function of the flagellar system come at a high energetic cost for the bacterium (2, 3). To save resources, bacteria use different mechanisms to prevent unnecessary flagellar expression. The human foodborne pathogen *Listeria monocytogenes* is the causative agent of listeriosis in humans (4). Although the number of cases of listeriosis is relatively low compared to other infectious diseases, the mortality among infected individuals is high (20%–30%) (5). Its ability to grow at very low temperatures (down to 0°C) and high salt concentrations makes *L. monocytogenes* a large problem for the food industry. The control of motility gene expression is complex in *Listeria monocytogenes,* with flagella synthesis being repressed at the temperature of the mammalian host (6–9). This requires a transcriptional repressor (MogR), which is rendered inactive by an anti-repressor (GmaR) at low temperatures. At higher temperatures, GmaR is inactivated and cannot bind MogR, thus allowing MogR to repress expression of motility genes. In addition, a large antisense RNA (AS-RNA) has been shown to repress expression of motility genes (10). The exact mechanism by which the AS-RNA acts is not known, but it involves direct interaction of the AS-RNA with the large motility transcript (10), possibly inducing RNA degradation or blocking access of the ribosome, as has been shown for other antisense RNAs in *Listeria* (11). The flagellar motility gene cluster is downregulated under stress conditions, such as high salt concentrations and blue light (12–15). The stress-mediated repression of motility genes is believed to be exerted by the stress-sigma factor σ^B^ (SigB) (16, 17), and expression of the AS-RNA is exclusively controlled by SigB (10).

The activity of the stress-sigma factor SigB is controlled through a complex signal transduction cascade involving several proteins (18–22). At the top of the regulatory hierarchy lies a large protein complex known as the stressosome. In the absence of stress, the stressosome consists of at least three different proteins, RsbR, RsbS, and RsbT, with a stoichiometry of 40:20:20, respectively. The stressosome integrates different external stress signals (e.g., salt, acid) to ultimately induce SigB activity. When the bacterium encounters stress, RsbT acts as a kinase, phosphorylating RsbR and RsbS proteins, which induces the release of RsbT from the stressosome. RsbT is then able to transiently interact with RsbU, promoting the phosphatase activity of the latter. RsbU dephosphorylates RsbV (an anti-anti-sigma factor), which allows RsbV to interact with and sequester RsbW (an anti-sigma factor). SigB is thus freed to interact with the RNA polymerase and initiate the transcription of stress-regulated genes. Once the stress has been dissipated and elevated SigB activity is no longer required, the stressosome needs to be reset to a sensing-proficient state. Ultimately, this requires resetting of the phosphorylated stressosome components by the phosphatase RsbX. Strains lacking RsbX cannot reset the stressosome and show high SigB activity even under non-stressed conditions (23–25).

While most research examining the role of SigB has been conducted in strains lacking SigB, in this study, we have focused on the effect of constitutive SigB activity on motility and motility gene expression. A strain showing high SigB activity (Δ*rsbX* mutant) is non-motile due to downregulation of the motility genes. A combined action of the AS-RNA (SigB regulated) and MogR (SigA regulated) mediates repression of the motility genes. We present evidence that the repressive effect of SigB is mediated via the stressosome since RsbR mutants giving constitutive SigB activity mirror the Δ*rsbX* mutant strain. The data point to an essential role for the stressosome in modulating motility appropriately during conditions of light and salt stress.

## MATERIALS AND METHODS

### Bacterial strains, strain constructions, plasmids, and oligonucleotides used in the study

Bacterial strains, plasmids, and oligonucleotides used in this study are shown in Tables 1 and 2.

**TABLE 1:** Strains and plasmids used in this study

Strain or plasmid	Reference
*Listeria monocytogenes* strains	
EGD-e (wild type)	(26)
EGD-e Δ*sigB*	(25)
EGD-e Δ*rsbX*	(25)
EGD-e Δ*rsbX* +pIMK3-rsbX	(24)
EGD-e Δ*rsbX* Δ*sigB*	(24)
EGD-e Δ*mogR*	(8)
EGD-e Δ*mogR* Δ*gmaR*	(9)
EGD-e Δ*gmaR*	(9)
EGD-e Δ*mogR* Δ*rsbX*	This work
EGD-e ΔP1_AS-RNA_	(10)
EGD-e Δ*rsbX* P1_AS-RNA_	This work
EGD-e Δ*rsbX* swarm outer #1	This work
EGD-e Δ*rsbX* swarm outer #2	This work
EGD-e Δ*rsbX* swarm inner #2	This work
EGD-e Δ*gmaR+* pIMK3 *gmaR*	This work
EGD-e+pIMK3	This work
EGD-e Δ*rsbX* +pIMK3	(24)
EGD-e+pIMK3-*gmaR*	This work
EGD-e *rsbR1*(T241A)	(27)
EGD-e *rsbT* (N49A)	(28)
EGD-e *rsbS* (S56A)	(27)
EGD-e Δ*rsbR* (Δ*rsbL* Δ*rsbR2* Δ*rsbR3* Δ*rsbR4*)	(27)
EGD-e *rsbR* (T175A)	(28)
EGD-e *rsbR1*(T209A)	(27)
EGD-e *rsbR1*(T241A)+pIMK3	This work
EGD-e *rsbR1*(T241A)+pIMK3-*gmaR*	This work
EGD-e *rsbR1*(T241A)+pIMK3-*rsbX*	This work
EGD-e *rsbR1*(T209A)+pIMK3	This work
EGD-e *rsbR1*(T209A)+pIMK3-*gmaR*	This work
EGD-e *rsbR1*(T209A)+pIMK3-*rsbX*	This work
*Escherichia coli* strain	
S 17.1	(29)
Plasmids	
pMAD-*rsbX*	(24)
pIMK3-*rsbX*	(24)
pIMK3	(30)
7pIMK3-*gmaR*	This work

**TABLE 2:** Oligonucleotides used in this study

Oligonucleotide sequence (5′−3′)	Target
Oligonucleotides used for cloning	
AAAGGATCCAGGAGGAAAAATATGCGGCCGTTAATTTCGATTTG	*gmaR lmo0688* fwd1^*a*^
ACGCCTGCAGTTTCTTCTGTCATATTACTGGCCTCCTA	*gmaR lmo0688* rev1^*b*^
Oligonucleotides used for sequencing	
ACGAGTGACAACAACAACC	*gmaR lmo0688* forward
CGACAATTTCCGACATATAGCC	*gmaR lmo0688* reverse
ACGCCTGCAGTTTCTTCTGTCATATTACTGGCCTCCTA	*gmaR lmo0688* rev2
CGAACCGAATCAGCGAGGCAATGG	*gmaR lmo0688* seq fwd
AAAGGATCCAGGAGGAAAAATATGCGGCCGTTAATTTCGATTTG	*gmaR lmo0688* fwd1
ACGCCTGCAGTTTCTTCTGTCATATTACTGGCCTCCTA	*gmaR lmo0688* rev1
Oligonucleotides used for RT-qPCR	
GCAGTACAATCTTCTAACGGTTC	*flaA* fwd
GCTTGGATGCTTACTTGAGTAG	*flaA* rev
GCTTCCATTCAGCTAGTCTGA	tmRNA fwd
CCTCGTTATCAACGTCAAAGC	tmRNA rev
GCATATTCGAAGTGCCATTGC	*lmo2230* fwd
GGTGAATAAGACAAACTTTCAGGTG	*lmo2230* rev
GCAACCACTTATTACTGCTTG	*fliP* fwd
GGATCTTCTCCTTCTGCTTTCAA	*fliP* rev
CCTTGATATCTCTGAAATGCTCAG	*mogR* fwd
CCAGGCTTGTTTTTCGGAATATC	*mogR* rev
CCAACGGAACCCATTCAAATG	*lmo0688* fwd
CGTTTGTCGATTGCTTCTAGTT	*lmo0688* rev
Oligonucleotides used for Northern blot	
TAATACGACTCACTATAGGACGGGGTTAATTGTAGTGTGG	T7-*lmo0676* fwd
CCAAGCAGTAATAAGTGGTTGC	*lmo0676* rev
CGGCACTTAAATATCTACGAGC	tmRNA-U
CCTCGTTATCAACGTCAAAGCC	tmRNA-L

^*a*^ Restriction endonuclease *BamH*I sequence is underlined.

^*b*^ Restriction endonuclease *Pst*I sequence is underlined.

To generate *L. monocytogenes* Δ*mogR,* Δ*rsbX,* and Δ*rsbX,* ΔP1_AS-RNA_ mutant strains, the plasmid pMAD-Δ*rsbX* (shuttle vector pMAD containing the *rsbX* deletion construct [25]) was transformed by electroporation, using the settings 400 Ω, 25 µF, and 2.5 kV, into electrocompetent EGD-e, ΔP1 and Δ*mogR* cells. Selection of transformants, chromosomal integration and excision of the vector, and screening for bacterial mutant strains were done as previously described (31). The deletions were verified by PCR.

Generation of *gmaR* and *rsbX* complemented strains: primers *gmaR* fwd 1 and *gmaR* rev 1 were used to amplify *gmaR* by PCR. The plasmid pIMK3 and the purified PCR product were digested with the restriction endonucleases BamHI and PstI, and the PCR product was ligated into the plasmid using T4 DNA ligase to create pIMK3-*gmaR*. The accuracy of the sequence in the construct was verified by restriction endonuclease digestion and sequencing. Electrocompetent *E. coli* S 17.1 were electroporated with pIMK3-*gmaR* and pIMK3-*rsbX*, separately*,* and transformants were selected on Km LA plates (50 µg/mL). The resulting strains *E. coli* S 17.1+ pIMK3 *gmaR* and S 17.1+ pIMK3 *rsbX* were used for conjugation with *Listeria monocytogenes* strains EGD-e, Δ*rsbX,* Δ*gmaR, rsbR1*(T241A), and *rsbR1*(T209A). Five milliliters of overnight cultures of *E. coli* S 17.1+ pIMK3 *gmaR* and S 17.1+ pIMK3 *rsbX* grown in 1× LB were centrifuged at 7,200 RCF, at 23°C for 7 min and resuspended in 5 mL 1× LB. One milliliter of the suspension was centrifuged at 6,000 RCF, at 23°C for 7 min and resuspended in 1 mL BHI. Two hundred microliters of overnight cultures of the *L. monocytogenes* strains grown in BHI was mixed with 200 µL of the resuspended *E. coli* S 17.1+ pIMK3 *gmaR* and S 17.1+ pIMK3 *rsbX* strains (separate mixtures), and 200 µL of the mix was put onto a cellulose nitrate filter (Sartorius Biotech) placed on BHI plates and incubated at 37°C overnight. Bacteria were washed from plates with 1.5 mL BHI, centrifuged at 6,000 RCF, 23°C for 7 min, and resuspended in 200 µL BHI. One hundred microliters was spread on BHI plates (1.5% agar [wt/vol]) containing 50 µg/mL Km, 50 µg/mL nalidixic acid sodium salt, and 10 µg/mL colistin and incubated at 37°C overnight. Transconjugants were restreaked several times on selective plates.

### Motility measurements

Two microliter aliquots of overnight bacterial cultures, all at approximately similar optical densities (ODs), were placed on BHI plates containing 0.3% (wt/vol) agar at 23°C. Swarming was quantified by measuring the diameter of the colonies once every day at the same time point for 4 days. Two measurements of the diameters, perpendicular to each other, were performed for each colony and the average diameter was calculated. The inoculum diameter was subtracted from the colony diameter value. For motility measurements of strains harboring the isopropyl β-D-thiogalactopyranoside (IPTG)-inducible plasmid pIMK3, 1 mM IPTG was added to the BHI agar plates. When monitoring the motility of bacterial strains subjected to osmotic stress, NaCl was added to the plates to a concentration of 0.5 M.

### Whole genome sequencing of bacterial strains

From glycerol stocks stored at −80°C, WT (EGD-e) and Δ*rsbX* bacterial strains were streaked onto BHI agar (1.5% [wt/vol]) plates to obtain single colonies. Single colonies were stabbed into motility agar plates (BHI+0.3% agar [wt/vol]) and allowed to swarm at 23°C for 4 days. Bacteria from the inner and outer regions of the swarming colonies were streaked onto BHI agar (1.5% [wt/vol]) plates and then prepared as glycerol stocks and kept at −80°C. Four single Δ*rsbX* bacterial colonies originating from four separate outer swarming regions, as well as “pre-swarming” Δ*rsbX* and EGD-e parental bacterial colonies (that had not been swarming), were separately inoculated and grown in BHI at 37°C overnight. Genomic DNA was prepared from these cultures using the Qiagen DNeasy Blood & Tissue Kit according to the protocol for Gram-positive bacteria and sent to Microbes NG, Birmingham, UK, for whole genome sequencing.

### RNA sequencing

WT (EGD-e) and ∆*rsbX* bacterial strains were grown in BHI at 23°C under ambient laboratory lighting conditions until OD_600_ = 0.85. Bacterial cultures were mixed 5:1 with Stop solution (5% phenol in ethanol) and collected by centrifugation. The pellets were resuspended in Disruption solution (10% glucose, 12.5 mM Tris-HCl, pH 7.6, and 5 mM EDTA), and bacteria were disrupted with 0.1 mm glass beads using a FastPrep grinder (MP Biomedicals) in the regime: 45 s + 30 s, speed 6.5. RNA was isolated by mixing the lysates with TRI Reagent Solution (Thermo Fisher) and chloroform, followed by centrifugation. The aqueous samples were thereafter subjected to two additional chloroform extractions. RNA was precipitated with isopropanol, and the pellets were washed with 80% ethanol and dissolved in RNase-free water. RNA was treated with DNase I (Roche) and purified on RNeasy MinElute columns (Qiagen). The cDNA libraries were prepared using the TruSeq Stranded mRNA kit (Illumina) and sequenced on the MiSeq Sequencing System (Illumina) in a 2 × 76 bp paired-end mode. The sequencing reads were aligned to the *L. monocytogenes* EGD-e genome (NC_003210) with the Bowtie 2 aligner (32), and the differential expression analysis was performed using edgeR software (33). The sequencing data are deposited in NCBI’s Gene Expression Omnibus (34) under accession number GSE310143. Data are from three independent biological replicates of each strain.

### Protein extraction

For protein extraction using mutanolysin (Fig. 3A through C), to generate whole-cell extracts, overnight bacterial cultures were grown in BHI (+1 mM IPTG where the inducible plasmid pIMK3 was used) and diluted to an OD_600_ of 0.05 and grown at 23°C in BHI (+1 mM IPTG where pIMK3 was used). Five milliliters of bacterial cultures were harvested after various time points as indicated. For static conditions, the overnight cultures were diluted into five separate cultures, where each culture was allowed to grow statically for the time indicated. The static cultures were gently vortexed before samples were withdrawn. For experiments with swarmer colonies, the cultures were grown until an OD_600_ of 0.9. The pelleted bacterial cells were resuspended in a buffer containing 30 mM Tris-Cl, pH 8, 50 mM NaCl, and 5 mM EDTA. The amount of buffer was adjusted to equal 5 mL of bacterial culture at an OD_600_ of 1 = 250 µL resuspension buffer. Samples were centrifuged at 10,000 RCF at 4°C for 10 min, and pellets were suspended in acetone (amount adjusted to equal 5 mL of an OD_600_ of 1 = 250 µL), kept on ice for 10 min, and centrifuged at 18,000 RCF at 4°C for 10 min. Pellets were resuspended in a solution containing 50 mM Tris-Cl, pH 6.5, 200 U/mL mutanolysin (Sigma-Aldrich), 200 U/mL DNase I (Roche), 0.1 mg/mL RNase A (Invitrogen), and were incubated for 30 min at 37°C (volume was adjusted to equal 100 µL to OD_600_ of 1 of the bacterial culture). 6× Laemmli buffer was added to a concentration of 1× to the samples, which were stored at −20°C or used directly for SDS-PAGE.

For protein extraction using FastPrep (Fig. 5A and 7B), bacterial strains were inoculated overnight at 37°C in BHI (+50 µL/mL Km for experiments using pIMK3) and diluted to an OD_600_ of 0.05 in BHI (+1 mM IPTG in experiments where pIMK3 was used) and grown overnight at 23°C under dark conditions. In experiments where bacteria were subjected to salt stress, overnight cultures were diluted to an OD_600_ of 0.07 and grown under dark conditions at 23°C in 20 mL BHI + NaCl at a final concentration of 0.5 M for 7 h. Bacterial strains not subjected to stress were treated the same way, but no NaCl was added. To harvest the bacteria, volumes withdrawn were adjusted to equal the same ODs and centrifuged at 6,200 RCF, 10 min, 23°C. 300 µL of buffer A (200 mM KCl, 50 mM Tris-Cl, pH 8.0, 1 mM EDTA, 1 mM DTT + 1 protease inhibitor tablet (Roche)/10 mL buffer) was added. Bacterial cells were disrupted using a FastPrep machine (45 s + 30 s, speed 6.5) and centrifuged at 18,000 RCF for 15 min at 4°C. Two hundred fifty microliters of the supernatants was transferred to new tubes, centrifuged at 18,000 RCF for 15 min at 4°C, and again supernatants were transferred to new tubes. 6× Laemmli buffer was added to a concentration of 1× to the samples which were stored at −20°C or used directly for SDS-PAGE.

### SDS-PAGE and Western blot

Protein samples containing Laemmli buffer were boiled at 100°C for 5 min, loaded on 12% SDS-PAGE gels, and run at 80 V through the stacking gel and then at 180 V through the separation gel. Proteins were transferred to activated polyvinylidene difluoride membranes using the Bio-Rad Trans-Blot Turbo Transfer System. Membranes were blocked in 5% dry milk in phosphate-buffered saline with 0.1% Tween (PBS-T) at 4°C overnight and then rinsed in PBS-T before the primary antibody anti-FlaA was incubated (1:6,000 in 5% dry milk in PBS-T) for 1 h at room temperature. Washing of the membranes was done in PBS-T for 4 × 5 min. Secondary anti-rabbit horseradish peroxidase-conjugated antibody (as09602; Agrisera, Vännäs, Sweden) was incubated (1:10,000 in PBS-T) for 1 h at room temperature. Membranes were washed for 4 × 5 min in PBS-T and ECL Prime Western Blotting System (Amersham/GE Healthcare) was used for detection according to the manufacturer. Membranes were visualized using LAS 4000.

### RNA isolation and RT-qPCR

RNA isolation and RT-qPCR were basically following the protocol of (27) with some minor changes; *Listeria monocytogenes* strains were grown at 23°C in dark conditions for 4 h. In addition, 0.5 M NaCl was added to half of the cultures, and all the cultures were grown for an additional 20 min. One milliliter was withdrawn from the cultures and put into tubes containing 200 µL stop solution (5% phenol in 99% EtOH). Tubes were centrifuged at 11,300 RCF for 10 min at 4°C, and pellets were stored at −80°C. Pellets were suspended in 400 µL TRI Reagent (Invitrogen), and the suspension was transferred to tubes containing ~0.4 g glass beads. Bacterial cells were disrupted using a FastPrep machine (45 s + 30 s, speed setting 6.5) and then centrifuged at 18,000 RCF for 5 min at 4°C. RNA was isolated using the Direct-zol RNA Miniprep kit (Zymo Research) according to the manufacturer’s protocol for RNA purification including DNase I treatment. RNA was eluted with 25 µL water, and the concentration was determined using NanoDrop 1000 spectrophotometer. The RNA concentration was adjusted so all samples contained the same amount. cDNA synthesis was performed using the SuperScript IV first-strand synthesis system (Invitrogen) according to the manufacturer’s protocol, including removal of genomic DNA. cDNA was diluted 200 times, before RT-qPCR was performed using Quantitect SYBR Green PCR Kit (Qiagen) CFX Connect Real-Time PCR System (Bio-Rad) with the following parameters: 95°C for 15 min, 40 cycles of 95°C for 15 s, 53°C for 15 s, and 72°C for 30 s. Primer efficiencies for target genes had previously been tested using cDNA. tmRNA was used as a reference gene. Quantification values were calculated using Bio-Rad Maestro Software and the PfaffI relative expression formula (35, 36). Results were normalized to the expression of non-treated wild type and expressed as log_2_ fold relative expression ratios. Student’s *t*-test was used for statistical analysis.

### Atomic force microscopy (AFM)

Bacterial strains were grown overnight in BHI at 23°C, and 300 µL of the cultures were pelleted, washed several times in Milli-Q water, and then resuspended in 1 mL Milli-Q water. For bacteria isolated from plates, approximately 10 bacterial colonies were resuspended in 150 µL Milli-Q water. Fifty microliters of the suspensions was placed onto freshly cleaved mica and incubated for 10 min at room temperature, washed three times with fresh Milli-Q water, and air-dried before imaging. Imaging was done on a Nanoscope IIIa (Digital Instruments, Santa Barbara) Atomic Force Microscope using Tapping Mode. A probe was oscillated at its resonant frequency of approximately 300 kHz, selected by the Nanoscope software. Images were collected and shown in a surface plot of the height mode.

### RNA isolation for Northern blot

RNA isolation was basically performed as previously described (37), with some minor differences. Bacterial cultures were grown overnight in BHI at 23°C under dark conditions. Thereafter, they were diluted to OD_600_ of 0.7 in 25 mL BHI + 1 mM IPTG, grown for 4 h at 23°C under dark conditions, and then subjected to stress for 20 min by adding NaCl to a concentration of 0.5 M. Twenty milliliters of the cultures was withdrawn and added to tubes on ice containing 4 mL stop solution (5% phenol in 99% ethanol) and centrifuged at 7,200 RCF for 10 min at 4°C. Pellets were stored at −80°C. A wild-type strain not subjected to stress was included as a control. Pellets were resuspended in resuspension solution (10% glucose, 12.5 mM Tris-HCl (pH 7.5), and 5 mM EDTA) and ~0.4 g of 1 mm glass beads, and bacteria were disrupted using a FastPrep machine (45 s + 30 s, speed setting 6.5). The aqueous phase was resuspended in 1 mL TRI Reagent (Invitrogen), incubated for 5 min at RT, and extracted with 100 µL chloroform. After two additional extractions with chloroform/isoamyl alcohol (24:1), RNA was precipitated with 0.7 volumes of isopropanol. The RNA pellet was resuspended in 200 µL DEPC-treated water and DNase treated with 20 U of DNase I. After extraction, first with phenol/chloroform/isoamyl alcohol and then chloroform, the RNA was precipitated with 2.5 volumes of 99% ethanol and 1/10 volume of 3M sodium acetate (pH 4.6). The RNA pellet was resuspended in DEPC-treated water, and the concentration of the RNA was determined using NanoDrop 1000. RNA quality was verified on an agarose gel.

### Northern blot

Northern blot was performed as previously described (37), with some minor changes. After separation on a 1.2% agarose gel, containing 1× HEPES buffer (10× HEPES buffer: 0.2 M HEPES 50 mM NaAc, 10 mM EDTA, adjusted to pH 7) and 7.3% formaldehyde, for 4 h at 100 V, the RNA was transferred to a Hybond–N membrane (Amersham) by capillary transfer. Membranes were cross-linked with UV light and prehybridized in ROTI Hybri-Quick (Carl ROTH) solution containing 100 µg/mL salmon sperm DNA for 2 h at 65°C. The RNA probe was generated using the Ambion MAXIscript T7 *In Vitro* Transcription Kit (Invitrogen) according to the manufacturer, using ^32^P labeled UTP, and purified using Oligo Clean and Concentrator columns (Zymo Research). Twenty microliters of the purified RNA probe was added to 10 mL of ROTI Hybri-Quick (Carl ROTH) solution, preheated to 65°C, and subsequently added to the membranes in rotator tubes. Hybridization was carried out overnight at 65°C in a rotation oven. Membranes were washed three times at 66°C in preheated washing solution (2× SSC/0.1% SDS for 15 min, 1× SSC/0.1% SDS for 15 min, and 0.5% SSC/0.1% SDS for 15 min), exposed in a phosphorimager cassette, and developed using the Typhoon FLA9500 scanner.

### Rifampicin mutation frequency determination

Bacterial strains were grown in BHI at 37°C overnight, serially diluted, and plated on BHI plates (1.5% agar) containing 7 µg/mL rifampicin. After incubation overnight at 37°C, the frequency of rifampicin-resistant mutants was determined.

## RESULTS

### The non-motile phenotype observed in the Δ*rsbX* mutant is reversed by mutations in the SigB-activation pathway

**Fig 1:**
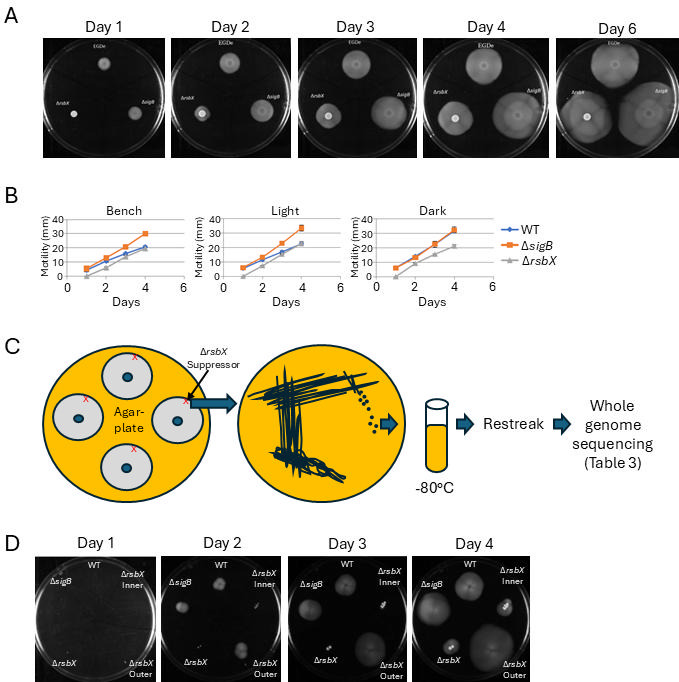
A Δ*rsbX* mutant strain regains motility by disrupting the SigB activation pathway. (**A**) Growth of indicated bacteria on BHI low-agar motility plates at 23°C on the bench for 6 days. (**B**) Measured motility (mm) of strains grown on BHI low-agar motility plates at 23°C on bench (left panel), under light conditions (middle panel), and in dark conditions (right panel), respectively, for 4 days. (**C**) Schematic drawing showing isolation of suppressor mutants used for whole genome sequencing. Δ*rsbX* mutant bacteria were grown on separate low-agar motility plates (only one plate is shown for simplicity). Bacteria growing at the outer edge of the colony after 4 days were picked, streaked to obtain single colonies, and re-streaked twice before whole genome sequencing. See Tables 1 and 3 for results of whole genome sequencing. (**D**) Growth of single bacteria on BHI low-agar motility plates at 23°C for 4 days. At day 0, overnight cultures of indicated bacteria were diluted to obtain single bacteria on the agar plate, and motility was followed over several days. Δ*rsbX* inner and Δ*rsbX* outer denote bacteria isolated from the inner part of the motility colony (inoculum) or the outer part of the motility colony (as depicted in panel C), respectively.

Previously, we observed that the Δ*rsbX* mutant was non-motile after 24 h incubation at 30°C on motility agar media, in contrast to the parental strain (24). Examining this further, we observed that the non-motile phenotype could be overcome by prolonged incubation (Fig. 1A). Strikingly, after 6 days of incubation, the Δ*rsbX* mutant had migrated as far as the WT. Quantifying the motility showed that the WT and the Δ*rsbX* mutant strains had both migrated equally far after 4 days of incubation at ambient laboratory light conditions, a condition where the bacteria face mild, non-lethal stress (14) despite the Δ*rsbX* mutant initially being non-motile (Fig. 1A and B). The reduced motility phenotype observed in the Δ*rsbX* mutant could be reversed by introducing a plasmid expressing *rsbX* (Fig. S1).

**TABLE 3:** Location of mutations in isolated suppressor mutants

				WT	Δ*rsbX*	Supp 1	Supp 2	Supp 3	Supp 4
Missense	aTg/aAg	M111K	*rsbT*						A
Missense	cCa/cTa	P42L	*rsbU*			T			
Nonsense	Gaa/Taa	E154*	*sigB*					T	
Deletion			Δ*dal* to *rsbT*				◊^*a*^		
Missense	gaT/gaA	D122E	*fliM*						A
Missense	Att/Ttt	I129F	*fliM*			T			
Missense	tgG/tgT	W212C	*fliM*					T	
Missense	cCa/cAa	P221Q	*fliM*				A		

^*a*^ ◊, 2,668 bp deletion spanning from *dal* to *rsbT* (Δ*dal-mazE-mazF-rsbR-rsbS-rsbT*).

We and others have observed that motility in *L. monocytogenes* was repressed when the bacterium was grown under ambient laboratory light conditions, and that this phenotype was dependent on the activity of the stress-sigma factor SigB (13, 14). Thus, as expected, motility was deregulated in the Δ*sigB* mutant, both when bacteria were grown at constant light or dark conditions, respectively (Fig. 1B). After 24 h exposure to ambient laboratory light conditions, the initial non-motile phenotype of the Δ*rsbX* mutant was altered, and the strain migrated at the same rate as the Δ*sigB* mutant and markedly faster than the WT strain (Fig. 1B, middle panel). Under constant dark conditions, all strains migrated equally fast, but the Δ*rsbX* mutant was unable to migrate as far as the other strains (Fig. 1B, right panel).

Previously, we observed that the Δ*rsbX* mutant grows more slowly than the WT and more slowly still compared to the Δ*sigB* mutant strain (24). The ability of the Δ*rsbX* mutant to move as fast as the Δ*sigB* mutant could indicate that the Δ*rsbX* mutant had acquired mutations in the SigB pathway, rendering SigB inactive. We therefore isolated cells from motility agar plates after 4 days of incubation, from the outer part of the Δ*rsbX* mutant colonies (where the bacteria were actively migrating, Fig. 1C). Four independent clones were isolated, and their genomes were sequenced. Each clone carried a mutation in the SigB-activation pathway: RsbT_M111K_; RsbU_P42L_; SigB_154*_; and a large deletion covering parts of the *sigB*-regulatory operon, respectively (Table 3). We also observed mutations in the *fliM* gene in all isolated suppressor mutants. However, when sequencing two WT isolates from the outer part of motility colonies, we observed that these strains also obtained mutations in *fliM* (*fliM*_H36D_ and *fliM*_D39E_, respectively). This shows that mutations also appear in the WT background during incubation on motility agar plates.

Although unlikely, it could be hypothesized that the mutations in the SigB-activation pathway were obtained already when the Δ*rsbX* mutant was generated and not selected for during growth on motility agar plates. We therefore made 10-fold serial dilutions of overnight cultures of different strains and spotted 10 µL of these dilutions onto motility agar plates, generating approximately one bacterium per strain. Incubating these at 23°C for 2 days showed that single colonies of the WT and the Δ*sigB* mutant migrated as shown previously (Fig. 1D). In contrast, single colonies of the Δ*rsbX* mutant did not migrate after 3 days but had started to migrate after 4 days (Fig. 1D). These data suggested that suppressor mutants were selected during growth on motility agar plates.

The Δ*rsbX* mutant did not show an intrinsically increased mutation frequency compared to the WT, as determined by quantifying the frequency of rifampicin-resistant mutants (Fig. S2).

### The absence of RsbX reduces motility gene expression

**Fig 2:**
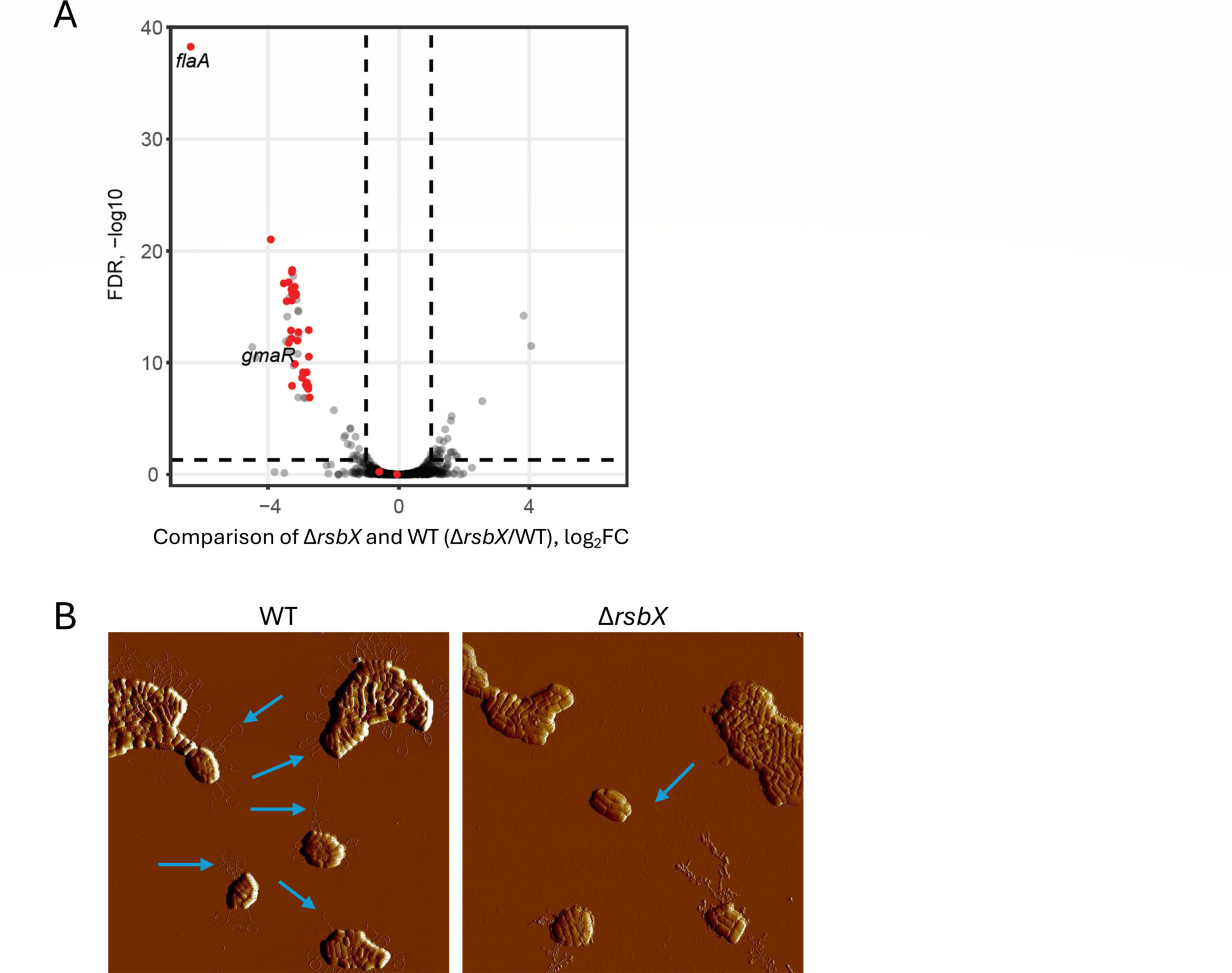
Expression of motility genes and formation of flagella are disrupted in the Δ*rsbX* mutant strain (see also Table S1). (**A**) Bacteria (WT and Δ*rsbX* mutant) were grown in BHI at 23°C under normal laboratory light conditions until OD_600_ = 0.85, when bacteria were harvested, RNA isolated, and subjected to RNA-seq. Volcano plot showing expression of genes in the Δ*rsbX* mutant strain in relation to the WT strain (log_2_ fold change [Δ*rsbX*/WT]). Circle denotes relative expression of a single gene. Circles in red show levels of motility genes (see also Table S1). (**B**) AFM images of WT and Δ*rsbX* mutant strains. Bacteria were grown in BHI at 23°C overnight before sampling. Arrows indicate flagellar structures. *n* = 3 (WT) and *n* = 3 (Δ*rsbX*), respectively.

Next, we sought to examine the impact of RsbX on global transcription. We thus compared gene expression by RNA-seq in the Δ*rsbX* mutant strain with the WT strain when grown in BHI medium at 23°C. RsbX has been shown to have a phosphatase activity, whose function is to dephosphorylate RsbR and RsbS, thereby resetting the stressosome for signal detection (24, 25). We did not exclude the possibility that RsbX could also have other targets. To examine this, we grew bacteria under ambient laboratory light conditions. By allowing both strains to be exposed to mild stress, indirect effects caused by hyperactive SigB, as observed in the Δ*rsbX* mutant, would be mitigated. In agreement with the observed motility phenotype (Fig. 1), our results show that motility genes were less expressed in the Δ*rsbX* mutant as compared to the WT (Fig. 2A; Table S1). These results were further corroborated when comparing WT and Δ*rsbX* mutant strains using an Atomic Force Microscope: in the Δ*rsbX* mutant strain, the flagella were either absent or aberrantly formed, as compared to the WT strain (Fig. 2B).

### Absence of RsbX dramatically reduces FlaA expression

**Fig 3:**
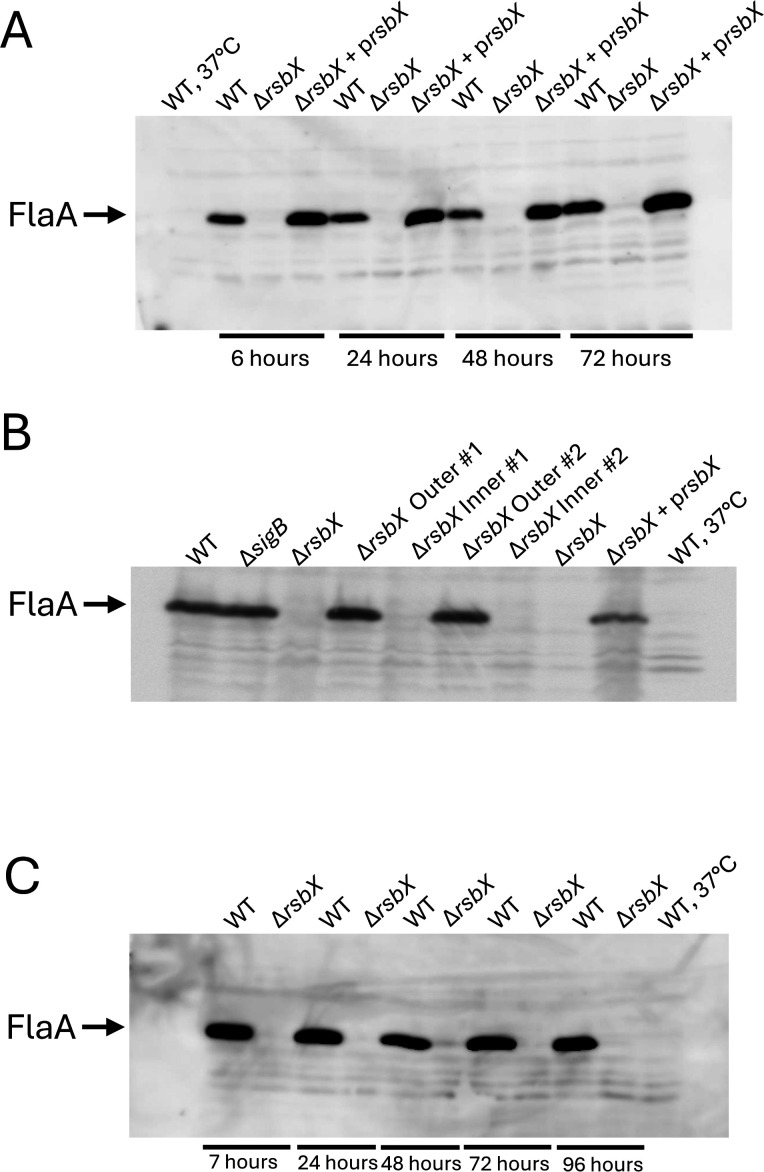
FlaA expression is abolished in the Δ*rsbX* mutant but is restored in the Δ*rsbX* mutant carrying *rsbX in trans* or in the suppressor mutants. (**A**) Indicated strains were grown in BHI at 23°C in shaking cultures for the indicated time points, when bacteria were harvested, protein isolated, and Western blot performed to detect FlaA. WT grown at 37°C was used as a negative control. (**B**) Growth of indicated bacterial strains in BHI at 23°C in shaking cultures until OD_600_ = 0.9 at 23°C when bacteria were harvested, protein isolated, and Western blot performed to detect FlaA. Δ*rsbX* “Outer” and Δ*rsbX* “Inner” denote Δ*rsbX* mutant bacteria isolated from the outer or the inner part of the motility-agar colony. #1 and #2 show two different isolates, respectively. (**C**) Growth of WT and Δ*rsbX* mutant strains in BHI at 23°C without shaking for the indicated time points when bacteria were harvested, protein isolated, and Western blot performed to detect FlaA.

The largest effect observed in strains lacking RsbX, when compared to the WT, was decreased motility and reduced expression of genes essential for motility. We thus decided to examine the role of RsbX and SigB on *L. monocytogenes* motility more closely. FlaA, or flagellin, is the main structural subunit of the flagellum and is one of the most highly expressed proteins in the bacterium at low temperatures. Our transcriptomic data, as well as the motility phenotype, indicated that expression of *flaA* was drastically reduced in the Δ*rsbX* mutant as compared to the WT. To examine this more closely, we followed FlaA levels over time in different strains grown in liquid media aerobically at 30°C. FlaA could not be detected in the Δ*rsbX* mutant but could be restored to WT levels by introducing *rsbX in trans* (Fig. 3A). Δ*rsbX* mutant bacteria isolated from the outer circle (but not the inner inoculum) showed a high expression of FlaA, in agreement with the increased motility observed in the outer circle mutant strains (Fig. 1D and 3B) and consistent with the derepressed motility observed in a Δ*sigB* background (Fig. 1A). In contrast, the non-motile bacteria isolated from the inner part of the motility agar colony and cultured as the “outer” colony did not express FlaA (Fig. 1D and 3B). Next, we were interested in examining whether static growth in a liquid culture would trigger mutant development. Despite extended incubation in non-shaking cultures, no expression of FlaA could be detected in the Δ*rsbX* mutant (Fig. 3C).

### Reduced FlaA expression observed in the Δ*rsbX* mutant can be restored by ectopic expression of GmaR

**Fig 4:**
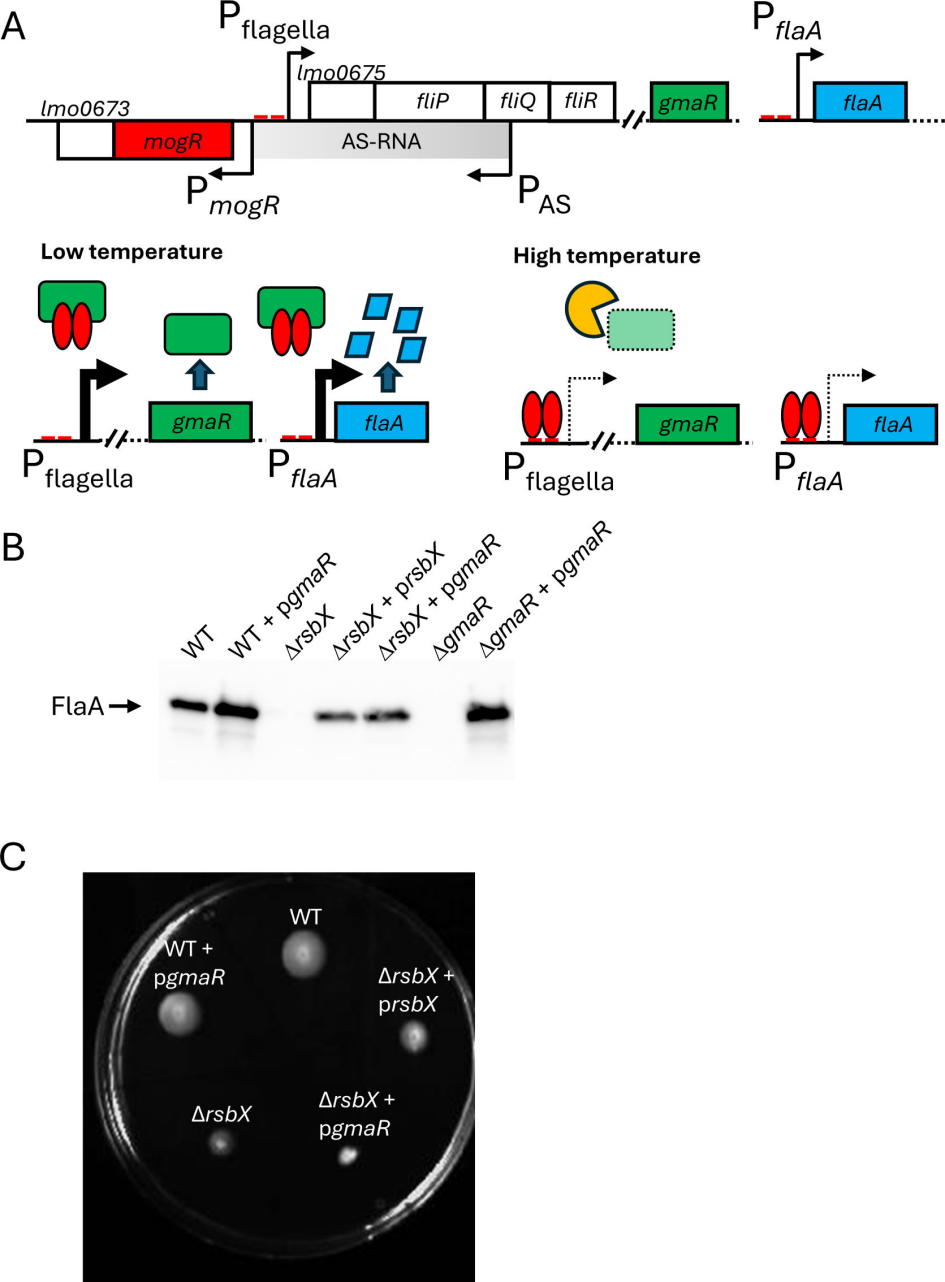
Expression of FlaA can be restored in the Δ*rsbX* mutant carrying *gmaR in trans*. (**A**) Upper panel: Schematic drawing of the regulatory part of the motility gene loci. P_fla_, P_AS_, P_mogR_, and P_flaA_ denote promoters responsible for expression of the large flagella operon, the antisense RNA, *mogR*, and *flaA*, respectively. Lower panel: Schematic drawing describing the GmaR/MogR regulation of motility gene expression. At low temperatures, GmaR sequesters MogR, thus preventing MogR-mediated repression of the flagella and the *flaA* operon. Motility genes are expressed. At high temperatures, GmaR is unstable, thus liberating MogR. MogR binds and represses expression of the flagella and the *flaA* operon. No expression of motility genes. (**B**) Expression of FlaA in indicated strains. Bacteria were grown in BHI at 23°C in shaking cultures until OD_600_ = 0.8, when they were harvested, protein isolated, and FlaA expression detected by Western blot. (**C**) Growth of indicated strains on BHI low-agar motility plates at 23°C for 48 h.

Expression of motility genes in *L. monocytogenes* is complex, involving several different regulators (Fig. 4A). The transcriptional repressor MogR binds to the operator region lying upstream of *lmo0675*, being the first gene of the large motility transcript. This transcript (*lmo0675* to *lmo0689*) harbors 15 genes encoding proteins that comprise large parts of the flagellum and flagellum motor complex. The anti-repressor GmaR counteracts the repressive effect of MogR at low temperatures by sequestering MogR (6, 7, 9). Within a mammalian host at 37°C, GmaR becomes inactivated, thus freeing MogR, which binds to its target operator sequences to repress motility gene expression. Transcription of *mogR* is partly controlled by the housekeeping sigma factor SigA (10). In addition, expression of motility genes has also been shown to be controlled by an AS-RNA, which acts by base-pairing with the first genes of the large motility transcript, and expression of the AS-RNA reduces the level of the long motility transcript (Fig. 4A) (10). Importantly, transcription of the AS-RNA relies solely on SigB-loaded RNA polymerase.

Transcriptomic data showed that expression of *gmaR* was reduced in the Δ*rsbX* mutant as compared to the WT (Fig. 2A; Table S1). The *gmaR* gene (*lmo0688*) is located on the long motility transcript, and its expression is controlled at the *lmo0675* promoter. Since GmaR is essential for inactivation of the repressor MogR, it could be hypothesized that reduced GmaR expression observed in the Δ*rsbX* mutant caused he non-motile phenotype and abolished FlaA expression. To examine this, we introduced a plasmid carrying *gmaR* into the Δ*rsbX* mutant, thus allowing expression of GmaR in the strain lacking RsbX. Strikingly, *gmaR* expression in the Δ*rsbX* mutant restored FlaA expression to levels similar to the WT (Fig. 4B). However, the Δ*rsbX* mutant expressing *gmaR in trans* was not more motile compared to the Δ*rsbX* mutant (Fig. 4C).

### RsbX-mediated regulation of motility genes requires MogR and AS-RNA

**Fig 5:**
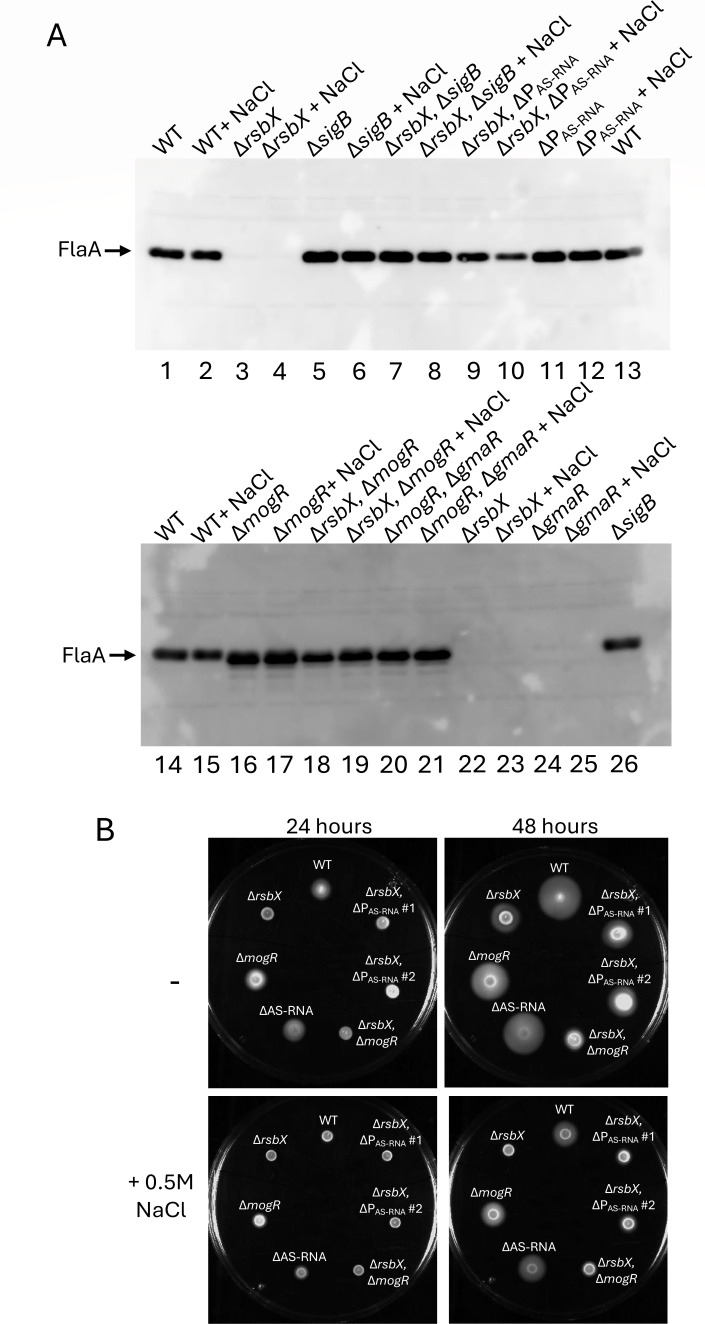
Disrupting AS-RNA or *mogR* expression in the Δ*rsbX* mutant restores FlaA expression but not motility. (**A**) Expression of FlaA in indicated strains. Bacteria were grown in BHI without (−) or with (+) supplementation of 0.5 M NaCl at 23°C in shaking cultures until OD_600_ = 0.8, when they were harvested, protein isolated, and FlaA expression detected by Western blot. (**B**) Growth of indicated strains on BHI low-agar motility plates without (−) or with (+) supplementation of 0.5 M NaCl at 23°C for 24 or 48 h.

Since expression of the motility genes is negatively controlled by both MogR and AS-RNA, we sought to examine the impact of these regulators on motility in a Δ*rsbX* strain. For this, we utilized FlaA as a proxy for motility gene expression. We also examined bacterial motility in non-stressed and stressed (0.5 M NaCl) conditions, since differential SigB activity is expected with changing osmolarity, and to exclude any non-SigB-mediated stress effects (38). As shown previously, the absence of GmaR completely abolished FlaA expression in a manner similar to the Δ*rsbX* mutant strain, and this effect was not altered by osmotic stress (Fig. 5A, lanes 3, 4, 22–25). When the Δ*rsbX* mutation was combined with a mutation removing the SigB-dependent promoter responsible for AS-RNA transcription (ΔP_AS-RNA_), the FlaA protein levels were restored to close to WT levels (Fig. 5A, compare lanes 2 and 3 with lanes 9 and 10). Likewise, in the Δ*rsbX*, Δ*mogR* mutant strain, FlaA levels were restored to near WT levels (Fig. 5A). External stress did not markedly affect the FlaA restoration observed in the Δ*rsbX*, ΔP_AS-RNA_, and the Δ*rsbX*, Δ*mogR* double mutant strains (Fig. 5A, compare lanes 18 and 19 and lanes 9 and 10 with lanes 22 and 23).

When analyzing the motility phenotype, the Δ*rsbX*, ΔP_AS-RNA_ and the Δ*rsbX*, Δ*mogR* mutant strains were not more motile compared to the Δ*rsbX* mutant strain (Fig. 5B). External stress (0.5 M NaCl) only slightly altered the motility phenotype. To examine if the Δ*rsbX*, ΔP_AS-RNA_ mutant strain was able to produce flagella, we examined flagella production of individual bacteria by AFM. The results suggest that the Δ*rsbX*, ΔP_AS-RNA_ mutant strain indeed can produce flagella, but that flagella assembly on the bacterial surface might be disturbed as compared to the WT strain (Fig. S3). Taken together, our results suggest that the effects of the Δ*rsbX* mutation on *L. monocytogenes* motility are mediated through a concerted action of both MogR and AS-RNA and thus dependent on the elevated SigB activity that is present in this genetic background.

**Fig 6:**
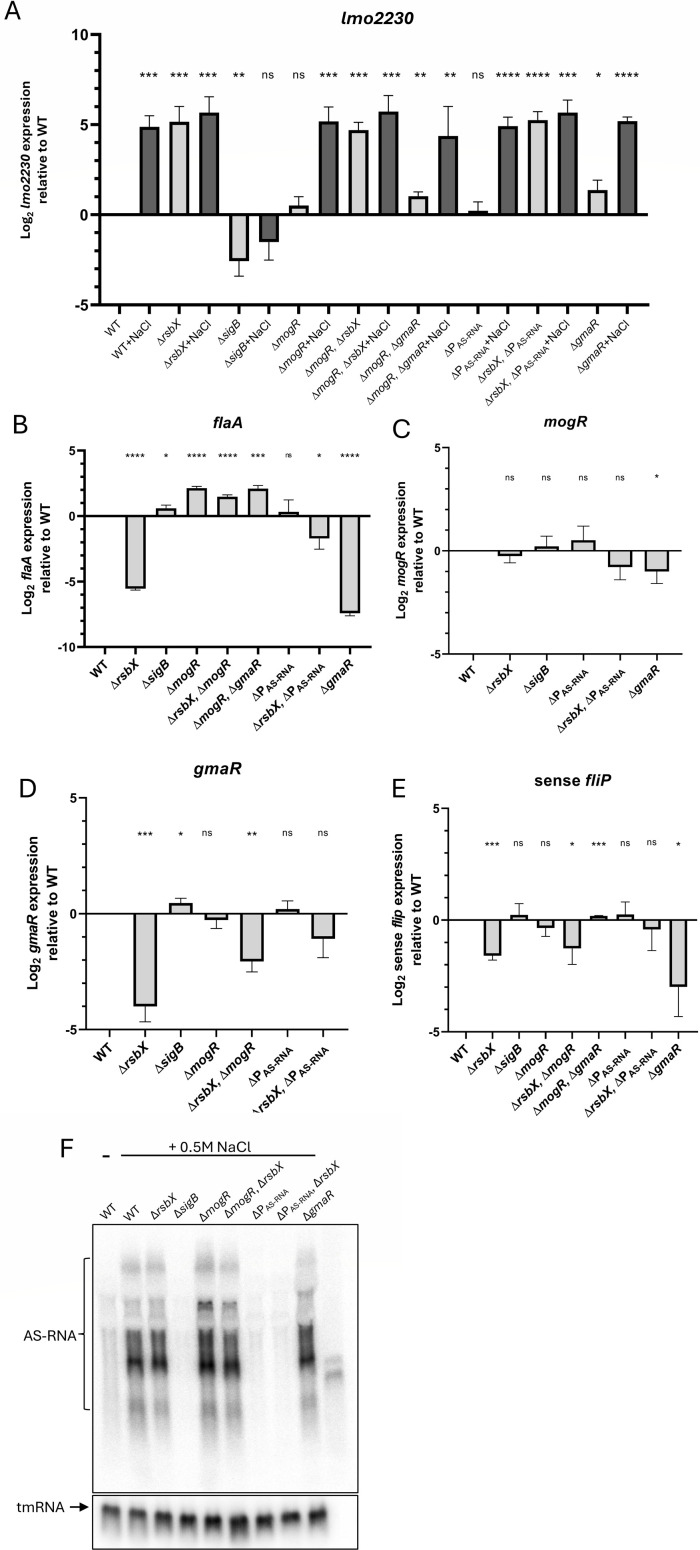
The reduced expression of flagella genes observed in the Δ*rsbX* mutant is increased by removing AS-RNA expression or MogR. (**A**) Indicated bacterial strains were grown in BHI without (−) or with (+) supplementation of 0.5 M NaCl at 23°C in shaking cultures until OD_600_ = 0.8, when they were harvested, RNA isolated, and expression of *lmo2230* was determined by RT-qPCR. (**B–E**) Indicated bacteria were grown in BHI at 23°C in shaking cultures until OD_600_ = 0.8, when they were harvested, RNA isolated, and expression of indicated mRNAs (B, *flaA*; C, *mogR*; D, *gmaR;* and E, *fliP*) was determined by RT-qPCR. (**F**) Indicated bacterial strains were grown in BHI supplemented with 0.5 M NaCl (except lane 1) at 23°C in shaking cultures until OD_600_ = 0.8, when they were harvested, RNA isolated, and expression of the AS-RNA was determined by Northern blot. tmRNA was used as a loading control (lower panel).

The involvement of MogR, GmaR, and AS-RNA in RsbX-mediated control of motility prompted us to examine expression levels of key players in different genetic backgrounds. First, we analyzed the ability of the different strains to activate SigB in response to osmotic stress. All strains, except the Δ*sigB* mutant strain, showed an increased expression of the highly SigB-regulated gene *lmo2230* when the bacteria were grown in the presence of 0.5 M NaCl (Fig. 6A). Strains lacking RsbX showed a constant high level of *lmo2230*, confirming the elevated SigB activity in this background regardless of the salt stimulus. Transcription of *flaA* mRNA mirrored the pattern observed for the FlaA protein: absence of RsbX or GmaR abolished *flaA* transcription. However, *flaA* transcription was restored to near WT levels in the Δ*rsbX*, ΔP_AS-RNA_ mutant strain, as well as the Δ*rsbX*, Δ*mogR* mutant strains, mirroring the FlaA protein expression levels (Fig. 5A and 6B). Transcription of *mogR* was almost unaffected in the different strains tested: neither the absence of RsbX, SigB, nor abolishing expression of the AS-RNA affected *mogR* expression, suggesting that transcription of *mogR* is almost exclusively directed from the SigA-controlled P2 promoter and that the AS-RNA transcript terminates before the P*mogR* (Fig. 6C). Transcription of *gmaR* was dramatically reduced in the Δ*rsbX* mutant strain, but somewhat restored in the Δ*rsbX*, Δ*mogR,* and the Δ*rsbX*, ΔP_AS-RNA_ mutant strains (Fig. 6D). Since *gmaR* is part of the large motility gene transcript (Fig. 4A), we were interested in examining whether the first genes of the transcript also showed a similar expression pattern. Analyzing expression of *fliP* in the different strains showed that its expression pattern mirrored the *gmaR* expression pattern (Fig. 6E). Finally, we examined the expression of the AS-RNA in the different strains. Since the AS-RNA has been shown to be present in different sizes, we examined its expression pattern by Northern blot (Fig. 6F, (14)). Expression of AS-RNA was strongly regulated by SigB as evidenced by strong induction in NaCl-exposed wild-type cells and the absence of transcript in the Δ*sigB* mutant. Absence of the SigB-controlled P1 promoter abolished AS-RNA expression, whereas the absence of MogR or GmaR did not affect AS-RNA expression (Fig. 6F). External stress (e.g., 0.5 M NaCl) did not markedly affect expression of *flaA, mogR, gmaR,* or *fliP*, respectively, in the different strains, suggesting that the effect we observed was channeled through the SigB pathway and not through any other stress-regulatory pathways (Fig. S4).

### The stressosome is involved in motility regulation

**Fig 7:**
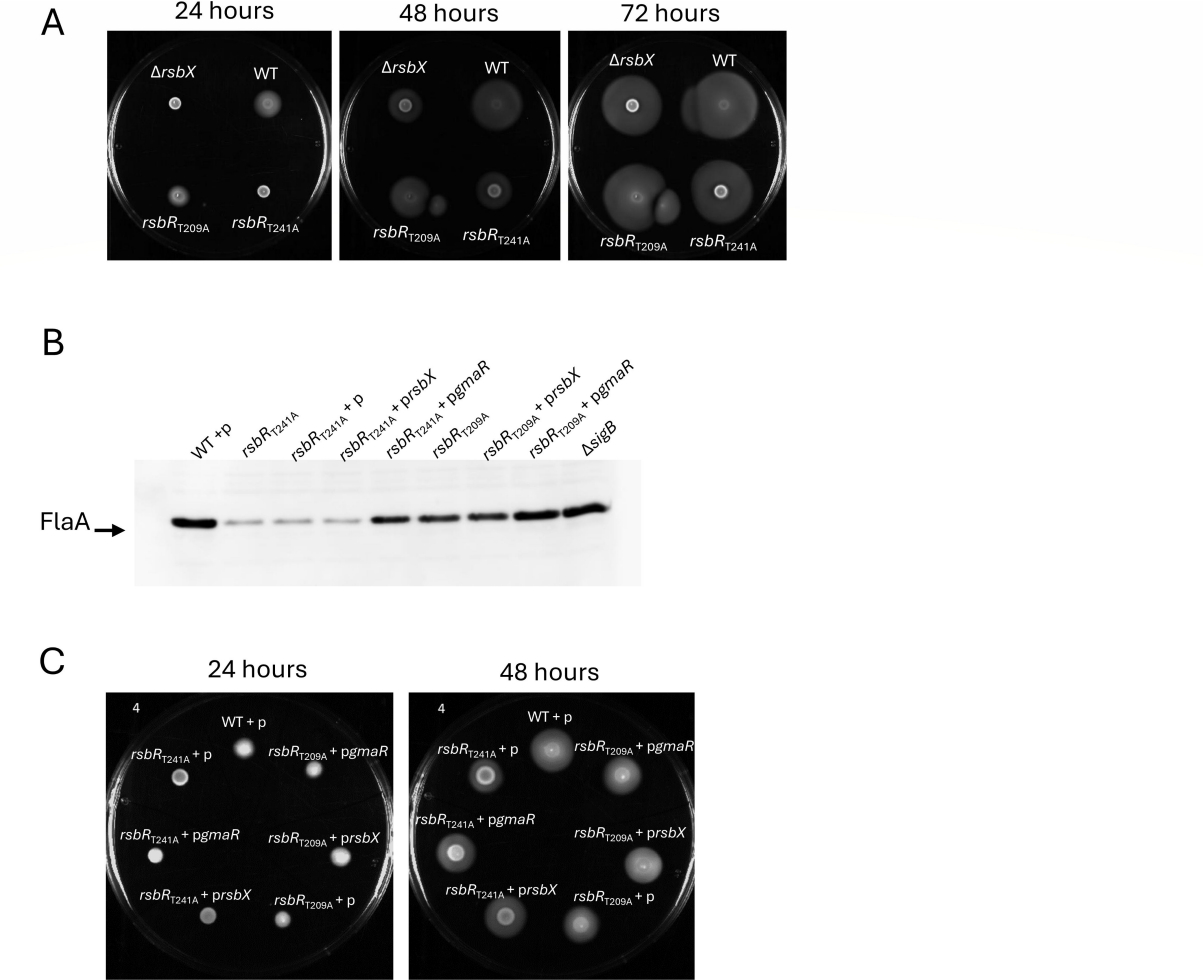
Motility and expression of FlaA is reduced in stressosome mutants increasing SigB activity but can be restored by expressing *gmaR*, but not *rsbX*, in *trans*. (**A**) Growth of indicated strains on BHI low-agar motility plates at 23°C for 24 or 48 h. (**B**) Expression of FlaA in indicated strains. Bacteria were grown in BHI at 23°C in shaking cultures until OD_600_ = 0.8 when they were harvested, protein isolated, and FlaA expression detected by Western blot. (**C**) Growth of indicated strains on BHI low-agar motility plates supplemented with 1 mM IPTG at 23°C for 24 or 48 h.

We and others have analyzed the role and function of the stressosome complex, which acts as a sensory hub that serves to integrate several stress cues into the SigB-activation pathway, thereby leading to the expression of hundreds of stress-regulated genes (19, 20, 39). One of the stressosome core subunits is RsbR (encoded by *rsbR/lmo0889*), which is thought to play a key role in stress sensing, likely through its N-terminal outward-facing “turrets,” although the mechanism of sensing remains to be revealed (40). Two RsbR mutants, *rsbR*_T209A_ and *rsbR*_T241A_, that abolished suspected phosphorylation sites of RsbR show elevated SigB activity in the absence of stress signals, in a manner similar to the Δ*rsbX* mutant strain (27). We were therefore interested in examining whether these mutants showed the same expression pattern and motility phenotype as the Δ*rsbX* mutant strain. The *rsbR*_T209A_ mutant showed reduced motility compared to the WT strain but did not display complete absence of motility during the first 24 h (Fig. 7A). In contrast, the motility phenotype observed in the *rsbR*_T241A_ mutant mirrored the motility phenotype observed in the Δ*rsbX* mutant strain: During the first 24 h of incubation, the *rsbR*_T241A_ mutant did not migrate on a motility agar plate (Fig. 7A) but after 48 h of incubation, the bacteria started to migrate, similar to the phenotype of the Δ*rsbX* mutant (Fig. 7A). Expression of FlaA was reduced in the *rsbR*_T241A_ mutant but could be restored to close to WT levels in an *rsbR*_T241A_ strain expressing GmaR (Fig. 7B). The *rsbR*_T209A_ mutant showed only a slight reduction in the level of FlaA, in line with the moderate motility phenotype (Fig. 7A and B). Interestingly, FlaA expression was not elevated in an RsbR_T241A_ strain expressing RsbX, indicating that overexpression of RsbX (phosphatase activity) cannot rescue the mutant phenotype. Although the level of FlaA was restored to close to wild-type levels, we were unable to observe increased motility in the *rsbR*_T241A_ strain expressing GmaR (Fig. 7C). Mutants lacking a functional SigB-activation pathway (*rsbT*_N49A_; *rsbS*_S56A_; and *rsbR*_T175A_, [27]) displayed increased motility as compared to the WT strain, thus mirroring a Δ*sigB* mutant strain (Fig. S5).

## DISCUSSION

In this work, we have studied the cross-talk between the SigB-regulated general stress response and motility in *L. monocytogenes*. The study arose from the initial observation that an Δ*rsbX* mutant strain had a non-motile phenotype on agar plates during the first 24 h of incubation but subsequently regained motility during extended incubation (24) (Fig. 1). Here, we show that the appearance of motility in the Δ*rsbX* mutant strain coincides with mutations that inactivate SigB, indicating a strong selection for such mutations. The mutations identified (Table 3) are predicted to negatively affect SigB activity through a premature stop codon in SigB (Suppressor 3), amino acid substitutions in the positively acting proteins RsbU (Suppressor 1) and RsbT (Suppressor 4), or a large deletion of the entire *sigB* operon itself (Suppressor 2). Bacteria lacking active SigB have a growth advantage compared to strains with functional SigB activity in non-stress conditions and, surprisingly, also under conditions of mild stress (21, 25, 41). This has been proposed to arise due to a trade-off between stress resistance and growth rate, both of which compete for cellular resources and energy (42). Excessive SigB activity appears to be suppressed during evolution as evidenced by the observation that the anti-sigma factor gene *rsbW* was not found to carry any premature stop codons among 22,340 sequenced *Listeria monocytogenes* isolates, and it has not been possible to construct an *rsbW* deletion strain (27). When *L. monocytogenes* encounters more severe (lethal) stress, SigB is essential, highlighting its importance for stress survival and its contribution to the long-term fitness of this pathogen (25). Interestingly, both the isolated suppressor mutants and two WT isolates from the outer part of the motility colonies harbored mutations in *fliM*. This suggests that the altered activity/levels of FliM were not causally linked to the restored motility phenotype in the Δ*rsbX* suppressor mutant strains.

The synthesis and operation of flagella come at a considerable cost for the bacterium (2, 3). To conserve energy during exposure to potentially lethal stress, bacteria have developed diverse mechanisms to regulate their flagella production (43). The control of motility gene expression is complex in *Listeria monocytogenes,* with flagella synthesis being repressed at the temperature of the mammalian host (Fig. 4). The exact mechanism by which the AS-RNA acts is not known, but it involves direct interaction of the AS-RNA with the large motility transcript (10), possibly inducing RNA degradation or blocking access of the ribosome, as has been shown for other AS-RNAs in *Listeria* (11). Expression of the AS-RNA is controlled exclusively by SigB, making the AS-RNA a good candidate to at least partially cause the motility phenotype observed in the Δ*rsbX* mutant strain. The data presented here show that the lack of *flaA* expression in the Δ*rsbX* mutant strain can be suppressed by either removing expression of the AS-RNA, deleting *mogR*, or by supplying *gmaR in trans* (Fig. 4 to 6). However, our data also suggests that MogR and AS-RNA-mediated repression of flagellar expression is not the sole cause of this phenotype since deletion of *mogR* or deletion of the P_AS-RNA_ in the Δ*rsbX* background does not reverse the loss of motility phenotype (Fig. 5). Flagella are produced and exported in the Δ*rsbX* ΔP_AS-RNA_ mutant strain but are not assembled correctly on the surface of the bacterium (Fig. S3), suggesting the possibility that increased SigB activity might interfere with the correct surface assembly of flagella.

**Fig 8:**
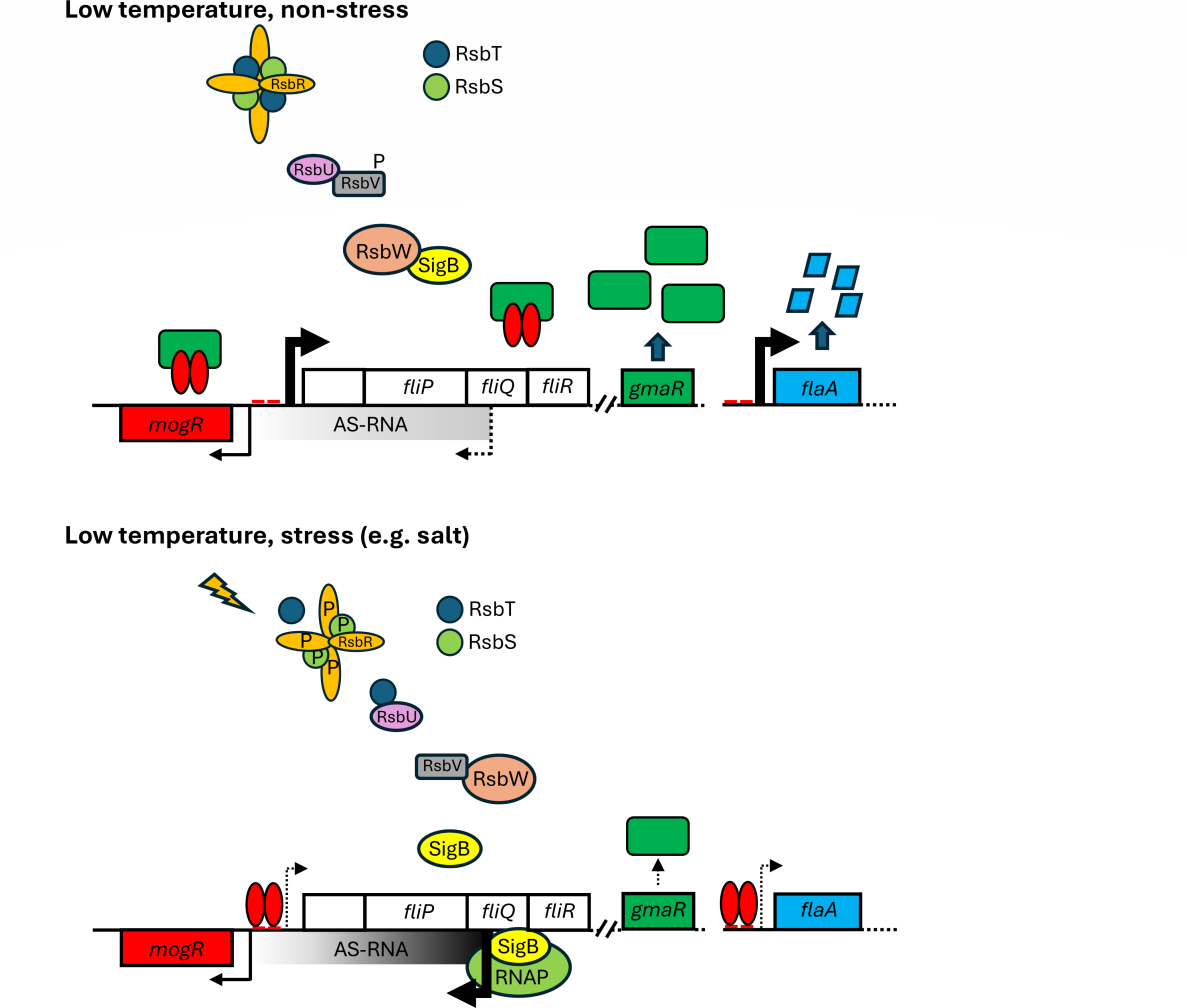
Model of stress-mediated regulation of the motility loci. At low temperature, non-stressed conditions, the stressosome is inactive, causing RsbW to sequester SigB. The AS-RNA is not expressed, allowing high expression of the large flagella operon (including GmaR) and FlaA. In case of stress, the stressosome becomes activated through a series of phosphorylations and dephosphorylations (P denotes a phosphor group), ultimately liberating SigB from RsbW. SigB associates with RNA polymerase and initiates expression of the AS-RNA, whose transcript terminates before the P*mogR* (faded gray bar). High expression of the AS-RNA leads to reduced expression of the large flagella operon. Since *gmaR* is part of the large flagella operon, the reduced expression of GmaR will liberate MogR, which can repress expression of the large flagella operon and FlaA. See text for further details.

Based on our results, we present the following model to account for the non-motile phenotype of the *ΔrsbX* mutant (Fig. 8): At low temperatures and under non-stressed conditions, the AS-RNA is expressed at low levels since SigB is in a non-active form, through its sequestration by RsbW. Under these conditions, the majority of MogR is sequestered by the antirepressor GmaR, allowing flagellar expression and motility. Under stress conditions, SigB becomes activated and can associate with RNA polymerase, thereby initiating expression of the SigB-dependent AS-RNA. The AS-RNA binds to and inactivates the large motility transcript rendering the bacteria non-motile. Since *gmaR* is part of the large motility transcript, the lower levels of GmaR will increase the amount of free MogR, which in turn can repress motility gene expression further. The synergistic action of MogR and AS-RNA leads to strong repression of bacterial motility gene expression. In the Δ*rsbX* mutant, this can only be reversed by mutating one or several genes in the SigB-activation pathway.

Stress-induced activation of SigB in *L. monocytogenes* involves the signal transduction through the stressosome, a high molecular weight sensory hub comprised of at least three proteins, RsbR, RsbS, and RsbT (20, 39, 44). Under adverse conditions, stress signals (e.g., blue light) are detected by the stressosome leading to RsbR and RsbS phosphorylation through the kinase activity of RsbT (20–22). This phosphorylation results in a conformational change that leads to the release of RsbT from the stressosome, allowing it to activate the downstream partner-switching events that ultimately lead to the release of SigB from its anti-sigma factor binding partner RsbW. The present study demonstrates that mutations in *rsbR (rsbR*_T209A_ and *rsbR*_T241A_), which disrupt the normal function of the stressosome, resulting in aberrantly high levels of SigB activity, also impair motility in a similar manner as the Δ*rsbX* mutant strain. It has been proposed that RsbR T209-PO_4_ and T241 can interact and that this interaction normally serves to suppress SigB activity (22). Substitution of either threonine residue with alanine was predicted to abolish this interaction and thereby give rise to increased SigB activity. The motility phenotype appears to be proportional to the level of SigB activation: The *rsbR*_T241A_ and the Δ*rsbX* mutant strain, which shows hyper-activation of SigB at non-stressed conditions, displayed the strongest repression of motility (Fig. 7; [27]). In contrast, the *rsbR*_T209A_ mutant strain, which shows a lower level of SigB activation at non-stressed conditions, was not as impaired in motility (Fig. 7).

Taken together, the findings in this study highlight the negative role that SigB has on motility in *L. monocytogenes*. Mutations that increase SigB activity suppress motility, and the only means of escape from this is the acquisition of *sigB* operon mutations that inhibit SigB activity. The mechanism of suppression appears to chiefly arise following stressosome signaling that induces the SigB-dependent AS-RNA that negatively affects expression of the large flagellar operon. The role of the GmaR anti-repressor, which is encoded in this operon, also appears to be crucial in allowing or preventing motility. It is clear that a trade-off exists between the two energy-intensive processes and under conditions of stress, with the focus prioritizing survival over motility.

## Data Availability

The sequencing data are deposited in NCBI’s Gene Expression Omnibus under accession number GSE310143.

## SUPPLEMENTAL MATERIAL

Supplemental figures aem.02539-25-s0001.pdf Fig. S1 to S5.

Table S1 aem.02539-25-s0002.xlsx Gene expression in the DrsbX mutant compared with WT.

ASM does not own the copyrights to Supplemental Material that may be linked to, or accessed through, an article. The authors have granted ASM a non-exclusive, world-wide license to publish the Supplemental Material files. Please contact the corresponding author directly for reuse.
