# Supplementary material for: Downregulation of motility during stress requires stressosome input in Listeria monocytogenes strain EGD-e
Source: Appl Environ Microbiol. 2026 Apr 3;92(4):e02539-25. doi: 10.1128/aem.02539-25 (PMC13101537; doi:10.1128/aem.02539-25)
Supplement: Supplemental figures — Fig. S1 to S5. [file aem.02539-25-s0001.pdf]

Figure S1

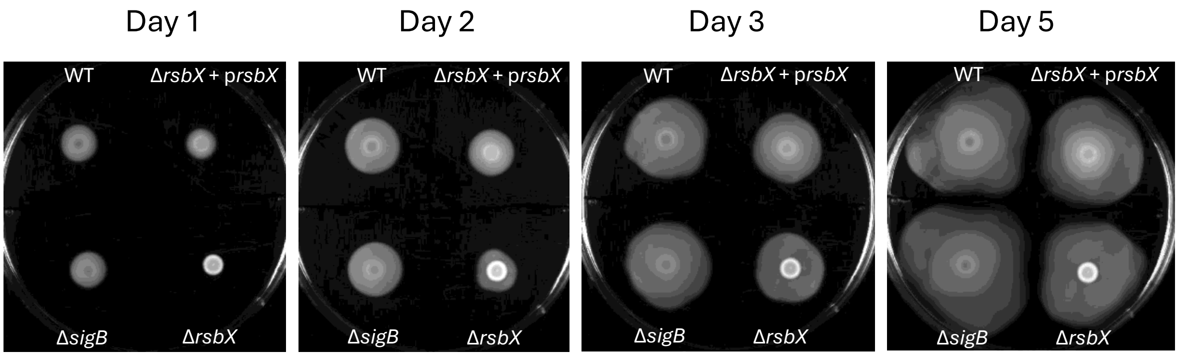

Figure S1. Motility can be restored in the Δ*rsbX* mutant expressing *rsbX in trans*. Growth of indicated strains on BHI low-agar motility plates supplemented with 1 mM IPTG at 23°C for 5 days.

Figure S2

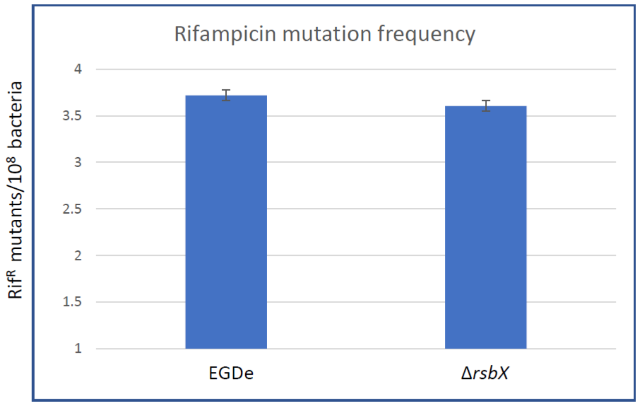

Figure S2. The mutation frequency is not increased in the Δ*rsbX* mutant as compared to the WT. Bacteria were grown overnight, before being plated on BHI-plates or BHI-plates supplemented with 7 µg/ml ug of Rifampicin and incubated overnight when the frequency of rifampicin resistant colonies was determined.

Figure S3

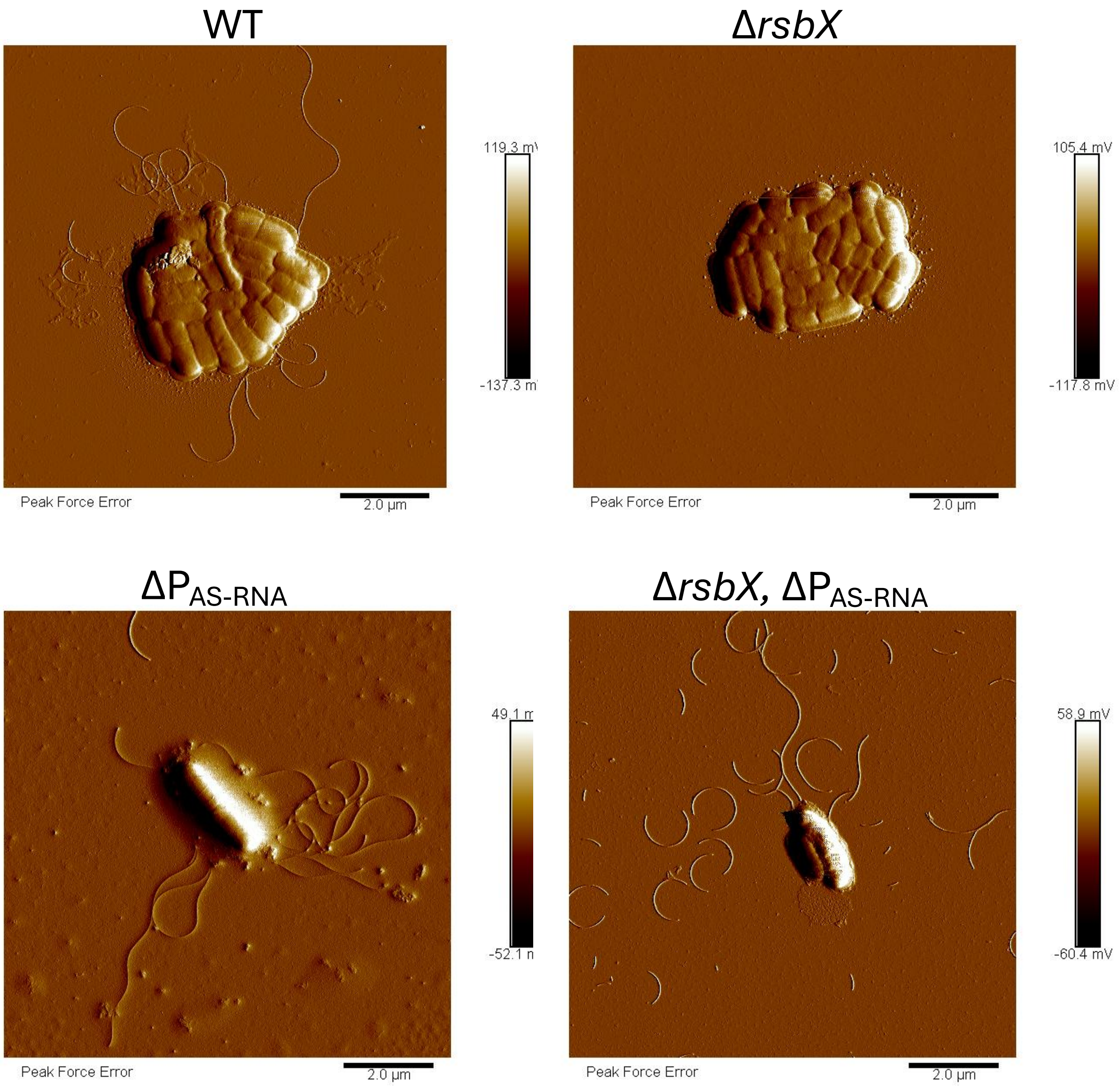

Figure S3. The production of flagella is restored in the Δ*rsbX,* Δ*rsbX*, Δ$P_{AS\text{-}RNA}$ mutant but its assembly on the bacterial surface is affected. Atomic Force Microscopy (AFM) images of indicated strains. Bacteria were grown in BHI at 23°C overnight before sampling. n=3 (WT); n=3 (Δ*rsbX*); n=2 (Δ$P_{AS\text{-}RNA}$) and n=4 (Δ*rsbX*, Δ$P_{AS\text{-}RNA}$), respectively.

Figure S4

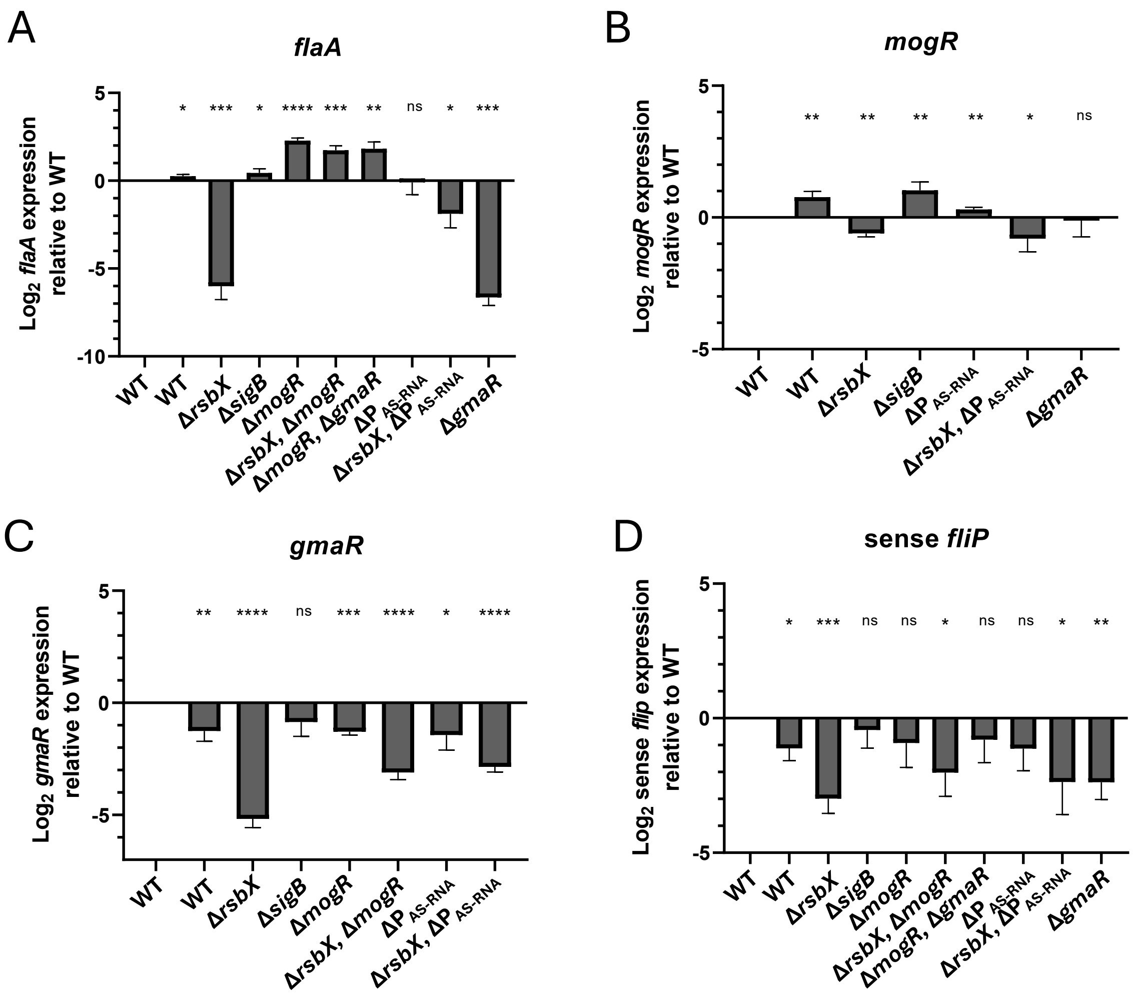

Figure S4. Indicated bacterial strains were grown in BHI supplemented with 0.5M NaCl at 23°C in shaking cultures until OD_{600} = 0.8, when they were harvested, RNA isolated and expression of indicated mRNAs (A – *flaA*; B – *mogR*; C – *gmaR* and D – *fliP*, respectively) was determined by RT-qPCR.

Figure S5

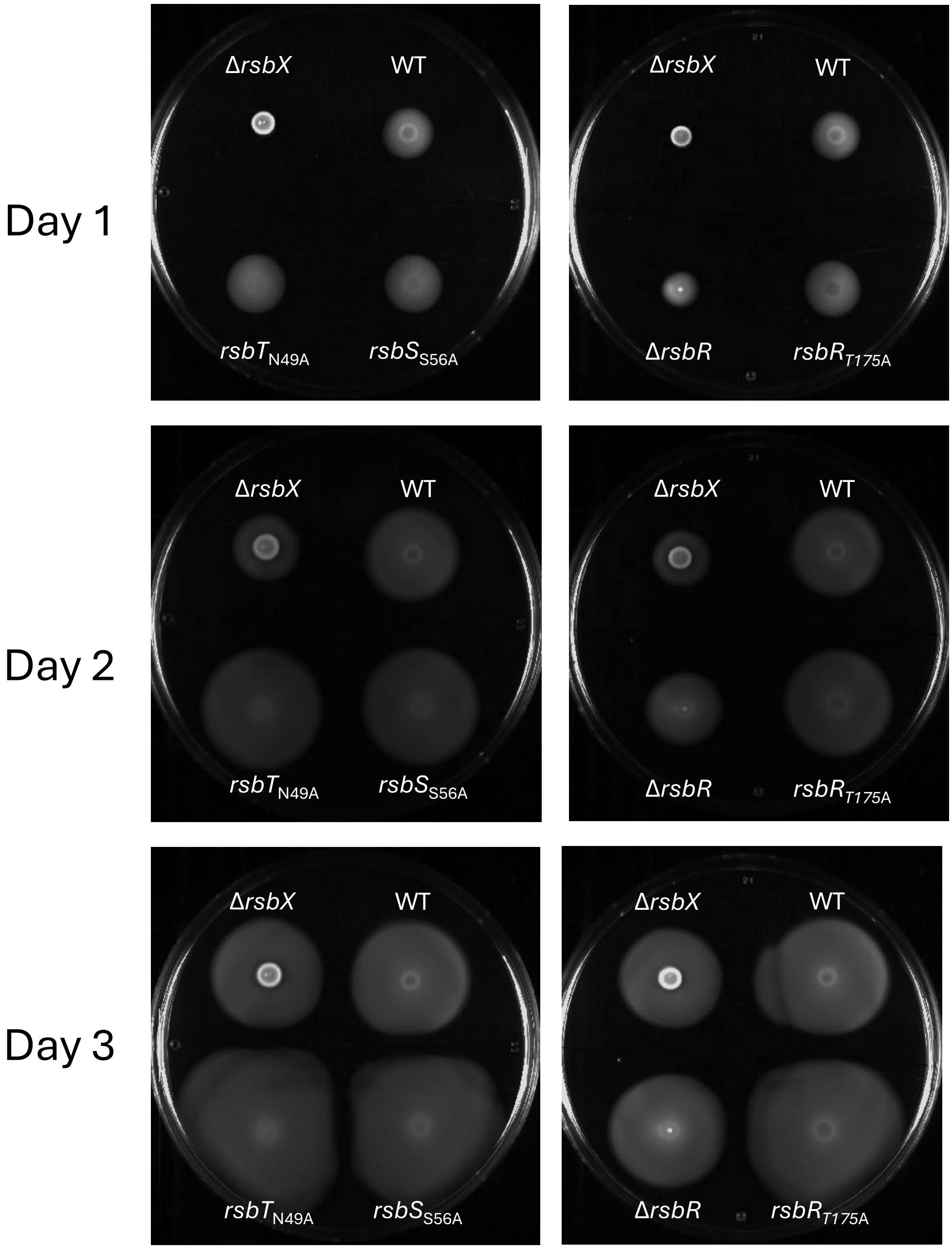

Figure S5. Mutants lacking a functional SigB-activation pathway show increased motility. Growth of indicated strains on BHI low-agar motility plates for 1 to 3 days at 23°C, respectively.
